# Gene Networks Involved in Hormonal Control of Root Development in *Arabidopsis thaliana*: A Framework for Studying Its Disturbance by Metal Stress

**DOI:** 10.3390/ijms160819195

**Published:** 2015-08-14

**Authors:** Stefanie De Smet, Ann Cuypers, Jaco Vangronsveld, Tony Remans

**Affiliations:** Centre for Environmental Sciences, Environmental Biology, Hasselt University, Agoralaan Gebouw D, 3590 Diepenbeek, Belgium; E-Mails: ann.cuypers@uhasselt.be (A.C.); jaco.vangronsveld@uhasselt.be (J.V.); tony.remans@uhasselt.be (T.R.)

**Keywords:** cadmium, copper, zinc, aluminium, phytohormones, lateral root, primary root

## Abstract

Plant survival under abiotic stress conditions requires morphological and physiological adaptations. Adverse soil conditions directly affect root development, although the underlying mechanisms remain largely to be discovered. Plant hormones regulate normal root growth and mediate root morphological responses to abiotic stress. Hormone synthesis, signal transduction, perception and cross-talk create a complex network in which metal stress can interfere, resulting in root growth alterations. We focus on *Arabidopsis thaliana*, for which gene networks in root development have been intensively studied, and supply essential terminology of anatomy and growth of roots. Knowledge of gene networks, mechanisms and interactions related to the role of plant hormones is reviewed. Most knowledge has been generated for auxin, the best-studied hormone with a pronounced primary role in root development. Furthermore, cytokinins, gibberellins, abscisic acid, ethylene, jasmonic acid, strigolactones, brassinosteroids and salicylic acid are discussed. Interactions between hormones that are of potential importance for root growth are described. This creates a framework that can be used for investigating the impact of abiotic stress factors on molecular mechanisms related to plant hormones, with the limited knowledge of the effects of the metals cadmium, copper and zinc on plant hormones and root development included as case example.

## 1. Root Development

**Figure 1:**
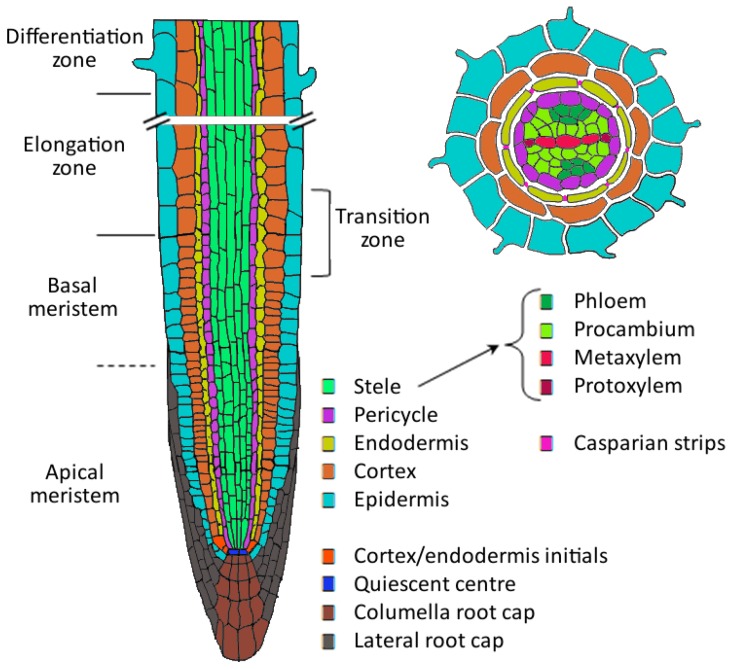
Organization of the *Arabidopsis* root. (**Left**): Longitudinal section through the root showing apical-basal polarity. Different cell types (each differently coloured) are arranged in cell files, forming concentric single-celled layers surrounding the central vascular tissue. Distinct developmental zones are formed along the growing root. Cell division occurs in the meristematic zone, especially the apical meristem. Cell division rate slows down in the basal meristem and cells start to elongate in the elongation zone. The boundary between meristematic and elongation zone is indicated as the transition zone. Cell differentiation occurs in the differentiation zone; (**Right**): Radial polarity in on a cross section of the differentiated root zone showing the formation of root hairs and Casparian strips (Based on Petricka *et al.*, 2012 [1] and Péret *et al.*, 2009 [2]).

Roots of *Arabidopsis thaliana* have a very simple cellular organization that can be described in a radial and an apical-basal polarity (Figure 1). At the radial centre of the root lays the diarch vascular bundle, consisting of two phloem and two xylem strands, the conductive tissues of the plant. The vascular bundle is surrounded by pericycle cells that can give rise to lateral root primordia. Next layers outwards are the endodermis, which forms a selective barrier for ions, and the cortex, that provides protection and mechanical support. The epidermis encloses the other tissues and contains the trichoblast cell lineage, which gives rise to root hairs [1,2,3].

Apical root growth is achieved by cell division and cell elongation. The root tip contains the root apical meristem (RAM), a region that consist of a set undifferentiated and dividing cells surrounding the quiescent centre (QC), a group of non-mitotically active cells. The QC is essential for maintaining the undifferentiated state of the surrounding stem cells, and for specifying the stem cell niche. The asymmetrical division of stem cells gives rise to self-renewing cells and daughter cells that in turn divide several times. Stem cells located above (shootward) and on the lateral sides of the QC will eventually differentiate into vascular, endodermal, cortical, epidermal and lateral root cap cells, whereas stem cells underneath the QC produce the columella root cap [1,2,3,4].

When the daughter cells age they will stop dividing and will start to elongate. This creates a visual border, or transition zone (TZ), in all cell lineages between the meristematic zone (MZ) and the elongation zone (EZ). Cell elongation pushes the stem cell niche at the root tip, which is protected by the root cap, further into the soil. Once cells have reached their full length they will differentiate, leading to a new zone in the root, the differentiation zone (DZ). In the DZ, root hairs appear in the trichoblast cells of the epidermis and casparian strips appear between endodermal cells [1,4].

**Figure 2:**
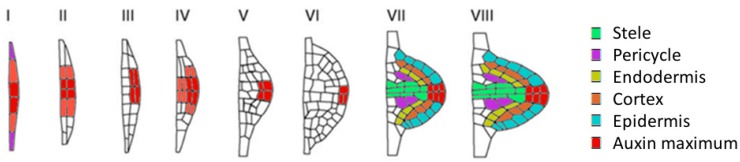
Lateral root development. (**I**) Lateral root initiation—Anticlinal division of lateral root founder cells in the pericycle; (**II**) Outer and inner cell layers are formed by periclinal divisions; (**III**) Periclinal divisions of the outer layer makes dome shape of the LRP is apparent (three-layered); (**IV**) As a result of periclinal divisions the primordium becomes four-layered; (**V**) After anticlinal divisions, the primordium begins to push through the cortex of the primary root; (**VI**) Different cell types are being formed; (**VII**) Lateral root meristem is established and primordium enlarges; and (**VIII**) Primordium is about to emerge after which the lateral root meristem will be activated (Based on Malamy and Benfey, 1997 [7]).

Lateral roots originate from mature pericycle cells positioned at either one of the xylem poles, but never at the phloem pool. Not all pericycle cells can form lateral roots, only those that have been primed before in the basal meristem. Primed cells are called lateral root founder cells and can become activated once a minimal threshold distance between the founder cell and the root tip is reached. Lateral root initiation (LRI), defined as the first division of the founder cells, occurs in the DZ [5,6]. A series of orchestrated periclinal and anticlinal cell divisions forms a lateral root primordium (LRP) in eight stages, described by Malamy and Benfey [7] (Figure 2). The emergence of the primordium through the parent root epidermis occurs mostly through cell expansion and involves cell wall remodelling enzymes that facilitate the separation of parental root cells. The LRP themselves are not affected by the enzymes because of differences in cell wall composition: the pectins in the primordia are largely methylated, while those of the parental cell wall are demethylated. Once the lateral root has emerged, its meristem is activated, and further growth results in a similar radial and an apical-basal polarity as described above [2,8].

## 2. Auxin

Auxin biology is one of the oldest fields of experimental plant research. When Charles Darwin studied phototropism, he noted that plants tend to orientate themselves for optimal growth and development and hypothesized that “something” was being moved from the root tip to the shoot, enabling it to bend [9]. Later, Darwin’s experiments were expanded by Theophil Ciesielski’s research on gravitropism in roots [10]. Since then, numerous studies have been performed on this phytohormone. Auxin is involved in virtually all aspects of plant growth and development including embryogenesis, organogenesis, tissue patterning, tropic responses to light and gravity, maintenance of apical dominance, vascular formation, shoot organ formation, and lateral and adventitious root formation [11,12,13,14,15,16].

### 2.1. Auxin Transport

Auxin is mainly synthetized in source tissues like young leaves and cotyledons, and is transported passively to the root sink via the phloem. This is a rapid, nonpolar type of long-distance auxin transport. In the root tip, active transport directs auxin downwards to the QC, then through the lateral root cap and epidermis to the basal part of the meristem (transition zone). At the TZ the auxin flux is reoriented inwards, back into the stele. This system is referred to as the inverted fountain model [17,18]. The active transport of auxin, also called polar auxin transport (PAT), is slower than the passive, but is important for auxin distribution over shorter distances. This cell-to-cell PAT is based on the combined actions of specific influx, and efflux carrier proteins [14,19,20,21,22]. In addition to PAT, maintenance of the auxin gradient across the root meristem is supported by root-synthesized auxin [23]. PIN proteins (PIN-FORMED, referring to the pin-like shape of the plant due structural abnormalities in inflorescence axes, flowers, and leaves when auxin transport is inhibited [24]) are efflux transporters with specific membrane localization that are responsible for the directional intercellular auxin flow. The PIN family contains eight members (PIN1-8) in *Arabidopsis thaliana*, each having cell-type specific expression and intracellular localisation. Phosphorylation of PIN proteins can change the direction of their exocytosis onto the plasma membrane. The polar intracellular location of the PINs is maintained by auxin itself as it inhibits PIN recycling by endocytosis [14,18,25,26,27,28]. ABCB/PGP (ATP-BINDING CASSETTE SUBFAMILY B/P-GLYCOPROTEINS) transporters mediate non-directional auxin efflux, their location in the plasma membrane is mostly non-polar. The best-characterized transporters of this group are ABCB1/PGP1 (found in all root cells except the columella cells), ABCB4/PGP4 and ABCB19/PGP19 (only in endodermis and pericycle) [16,18]. ABCB4/PGP4 is an auxin concentration dependent influx/efflux transporter. In response to increased auxin levels it switches the transport direction from influx to efflux [29]. The auxin influx transporter AUX1 (AUXIN RESISTANT 1) is an amino acid permease-like protein that acts as a H^+^/IAA^−^ symporter. The *Arabidopsis* genome encodes four AUX1/LAX (LIKE AUX1) influx carriers: AUX1, LAX1, LAX2 and LAX3. AUX1 is required for LRI because of its role in basipetal auxin transport in the epidermis [21,30].

### 2.2. Auxin Reception and Signal Transduction

There are at least two classes of auxin receptors: (1) ABP1 (AUXIN BINDING PROTEIN 1) and (2) co-receptors resulting from the auxin-induced interaction of TIR1/AFBs (TRANSPORT INHIBITOR RESPONSE 1/AUXIN SIGNALLING F-BOX) and Aux/IAA (auxin/indole-3-acetic acid). Auxin binding to ABP1 (1) induces hyperpolarization of the plasma membrane via the modulation of ion fluxes, especially by stimulating H^+^ efflux and K^+^ influx, in a dose-dependent manner [20,31]. The binding of auxin also increases the number of PIN proteins on the plasma membrane and thus enhances the cell capacity of auxin efflux [20,31,32]. TIR1 and AFB (2) are F-box proteins that interact with proteins of the Aux/IAA family, leading to their ubiquitination by the SCF (Skp, Cullin, F-box containing complex) and proteasomal degradation. Aux/IAA is a family of transcriptional repressors that interact with the ARF (AUXIN RESPONSE FACTOR) transcription activators [33,34,35]. TIR1, AFB1, AFB2 and AFB3 can interact with up to 29 members of the Aux/IAA family, with different affinities for each other and for auxin, giving rise to diverse pathways of transcriptional control of auxin responsive genes [36].

Another F-box protein was recently identified as an auxin receptor: SKP2A (S PHASE KINASE ASSOCIATED PROTEIN 2A). SKP2A is expressed from late S phase to M phase and mediates the degradation of E2Fc and DPb, transcriptional repressors for a subset of cell cycle regulated genes. SKP2A is considered a positive regulator of cell division, whereas E2Fc and DPb are involved in the regulation of the balance between proliferation and endoreduplication together with other E2F and DP dimers [37,38,39]. Binding of auxin to SKP2A triggers its own degradation and enhances E2Fc and DPb degradation [39]. New F-box co-receptors may be discovered, since SKP2A is taxonomically much more distant from TIR1/ARB than COI1, another F-box protein that is involved in jasmonate signalling but is unable to bind auxin [34,40].

### 2.3. Auxin in Primary Root Development

**Figure 3:**
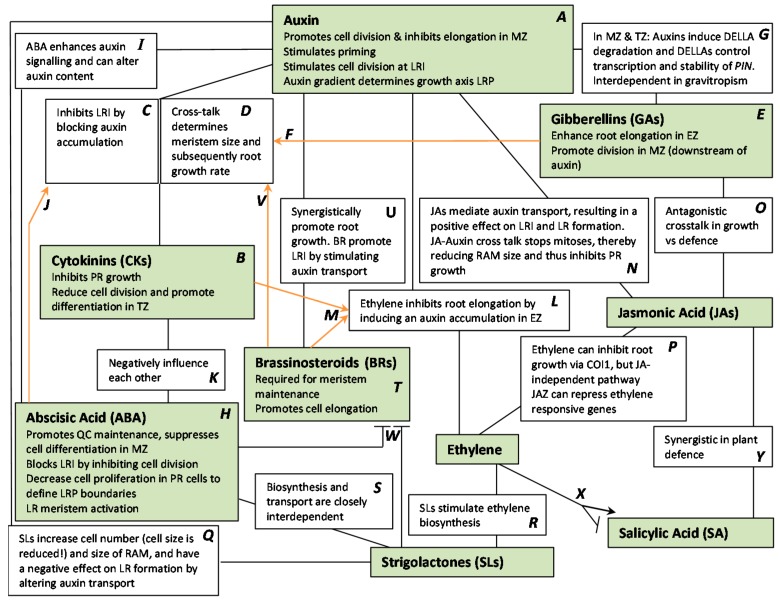
Schematic representation of the complex cross-talk network of hormones in root development. Individual effects of phytohormone on root development are described in the green boxes. Black lines with a black box describe a cross-talk between two phytohormones. Orange lines indicate that a certain phytohormone partakes in a cross-talk between two hormones. All information in this scheme is described more detailed in the text and can easily be found using the referral letters **A**–**Y**. **Abbreviations**: COI1 (CORONATINE INSENSITIVE 1), EZ (elongation zone), JAZ (JASMONATE ZIM-DOMAIN PROTEINS), LR (lateral root), LRI (lateral root initiation), LRP (lateral root primordium), MZ (meristematic zone), PIN (PIN FORMED), PR (primary root), QC (quiescent centre), RAM (root apical meristem), TZ (transition zone).

The result of active PAT is an auxin gradient in the MZ with a maximum in the QC. At the TZ the longitudinal auxin gradient is decreased as a result of the redirection of the flow towards the stele via PIN1 and PIN2 [41,42]. In the meristem the high levels of auxin stimulate cell division and inhibit cell elongation (Figure 3A). Auxin activates *PLT* genes (*PLETHORA*), which belong to the AP2 transcription factor family and act in a dose-dependent manner on the competence of cells to be maintained within the meristem [43]. Together with the hormonal crosstalk with cytokinins, involved in the regulation of the transition between the MZ and EZ through the regulation of SHY2 (SHORT HYPOCOTYL 2)/IAA3 repressor [44], the end of the auxin gradient defines the TZ as it allows cells to elongate.

### 2.4. Auxin in Priming of Lateral Root Founder Cells in the Basal Meristem

Auxin plays a prominent role in all aspects of lateral root formation, this is why interfering with auxin biosynthesis, transport, or response often leads to alterations in the lateral root formation [45]. The first step of lateral root formation is the priming of a founder cell in the pericycle lineage of the basal meristem. A peak of an oscillating and PIN1 and PIN2-dependent auxin signal in protoxylem strands coincides with the priming of the adjacent pericycle cells [1,30], oscillating expression of transcriptional regulators such as SHP1, SHP2, STK and AGL20 [46], and expression of the GATA23 TF (transcription factor) controlled by ARF7 and ARF19 via auxin-triggered degradation of IAA28 (Figure 3A) [47]. In addition to this endogenous process, external factors such as gravistimulation and mechanical bending can also induce lateral root formation. During gravistimulation PIN3 relocation redirects auxin flow towards the new lower side of the root tip and creates a lateral auxin gradient. Next, auxin is moved to expanding cells in the elongation zone via the combined actions of AUX1, PIN2, PGP1 and PGP19. The resulting auxin gradient inhibits cell elongation of epidermis cells, resulting in a bend that makes the primary root turn [1,48]. PIN1 relocation in a group of protoxylem cells at the convex side of the bend results in an auxin maximum in the protoxylem cells, determining a lateral root priming site [41]. This explains why lateral roots form at the convex side of bends [30] and forms the basis of the often used “root bending assay” for studying subsequent stages of LR development, as the location of a developing lateral root and the progression trough the stages are easily monitored [41,49,50].

### 2.5. Auxin in Lateral Root Initiation in the Differentiated Zone

Whereas LR founder cells are primed in the basal meristem, the actual LRI occurs in the DZ. The expression of CYCB1;1 is commonly used as a marker for LRI, because it is expressed during G2 and M phase and thus precedes the completion of the first cell division [51,52]. Auxin promotes LRI by alleviating KRP2-mediated repression of G1-to-S progression and regulating cell-cycle related genes such as cyclins, cyclin-dependent kinases (CDKs) [16,48]. Once cell division is activated, PIN1 proteins relocalize, thereby determining the growth axis and establishing an auxin gradient with its maximum in the tip of the primordium (Figure 2) [8,14]. When the growing primordium starts to emerge, LAX3 mediates the auxin influx in the outer endodermis and cortex cells, where it will induce cell wall modifying genes (Figure 3A) [8,53]. Once emerged, the meristem activation seems not to be controlled by auxin [54]. The role of auxin in lateral root development was more extensively reviewed by Lavenus *et al.* [55].

### 2.6. The Effect of Metal Exposure on Auxin in Root Development

Auxin is directly involved in root growth responses to environmental stresses, including exposure to excess metals (Table 1). Auxin transport in particular has been attributed a major role in these stress-induced changes. Excess copper (Cu) inhibits primary root elongation by decreasing *PIN1* expression, which resulted in a reduced meristematic cell division potential [56,57]. Interestingly, auxin distribution was solely modulated by PIN1 modulation, while PIN2 and AUX1 were unaffected. Although excess Cu induces oxidative stress, there was no apparent link between H_2_O_2_ accumulation and Cu-induced auxin redistribution [57]. PIN2 and AUX1 were involved in the aluminium (Al)-induced altering of auxin distribution, also resulting in inhibition of root elongation [58]. Cadmium (Cd) exposure altered the expression of *AUX1* and several *PIN* genes, and the *pin2-1* mutant failed to show the Cd-induced increased lateral root density [59]. The authors also found a decrease in IAA content, as a consequence of an increased activity of IAA oxidase and an altered expression of auxin biosynthetic and catabolic genes [59]. However, several other papers report an increased IAA concentration [56,60,61] as a result of Cd-mediated upregulation of the biosynthesic *NIT* (*NITRILASE*) gene [60,61]. Regardless of the effects of Cd on IAA contents, all reported an increased lateral root density during Cd exposure [56,60,61,62].

### 2.7. Auxin and Cross-Talk with Other Plant Hormones

Because of auxin’s prominent role in root development, numerous effects of other phytohormones on root development are the result of a crosstalk with auxin. In the following paragraphs, with each additional hormone its interactions with auxin and with the other hormones already discussed are added. This builds up a network of hormone actions and interactions that influence root development, which is visualized in Figure 3. Furthermore, we also refer to the review by Jung and McCouch [63] for an extensive overview of the genetic and hormonal control of intrinsic root development and the influence of various environmental factors on root development in different plant species.

## 3. Cytokinins

Cytokinins are N6-prenylated adenine derivatives involved in the control of several aspects of plant growth and development such as meristem activity, vascular differentiation, LRI, nodulation and response to biotic and abiotic stresses [64,65,66]. The major cytokinin forms in *Arabidopsis thaliana* are isopentenyladenine (iP) and trans-zeatin (tZ) [67]. Cytokinin is synthesised in roots, shoots and immature seeds, with tissue and organ specific patterns of expression of biosynthesis genes [68]. Cytokinins act as shoot-growth-promoting factors and negative regulators of root development. Exogenous cytokinin treatment inhibited root elongation [69], while reduction of endogenous cytokinin levels increased PR elongation [70,71].

In root cells cytokinin binds to the AHK4/WOL1/CRE1 (ARABIDOPSIS HISTIDINE KINASE 4/WOODEN LEG/CYTOKININ RESPONSE 1) receptor [1,72,73]. This induces autophosphorylation of the receptor and the phosphate group is subsequently transferred to members of the AHP (ARABIDOPSIS HIS-PHOSPHOTRANSFER PROTEINS) family, which migrate to the nucleus where they phosphorylate ARR (ARABIDOPSIS RESPONSE REGULATORS) TFs of type-A (ARR3-9, ARR15-17) and type-B (ARR1, ARR2, ARR10-14, ARR18-21). Phosphorylated type-B ARRs can activate downstream genes such as type-A *ARRs* and *CRFs* (*CYTOKININ RESPONSE FACTOR*). The phosphorylated type-A ARRs are negative regulators that can in turn repress type-B ARRs and response genes [74].

In parallel with primary root growth, cytokinins are also negative regulators of lateral root formation. Cytokinins inhibit LRI through AHK4 and AHP6 by downregulating *PIN* expression, thus preventing the establishment of the auxin maximum, thereby blocking the division of pericycle cells (Figure 3C) [75,76]. Auxin-cytokinin antagonism determines the final root meristem size and root growth rate (Figure 3D). While auxin promotes cell division, cytokinins reduce the number of dividing cells and inhibit root growth by promoting cellular differentiation in the TZ (Figure 3B) [1,44]. SHY2, an Aux/IAA-type repressor protein, is involved in this antagonism by influencing both auxin signalling and cytokinin synthesis. SHY2 negatively regulates *PIN* expression in the vascular tissue at TZ, thereby repressing auxin signalling and transport, and it downregulates *IPT5* expression, that is involved in cytokinin biosynthesis in roots [44,68]. Both hormones also control SYH2 abundance itself in opposite ways: cytokinins directly activate transcription of *SHY2* in the vascular tissue at the TZ through the AHK3–ARR1/ARR12 pathway [44], whereas auxin directs proteasomic degradation of the SHY2 protein via the SCF^TIR1^ ubiquitin ligase complex (Figure 3D) [77].

The primary root growth of Cu-stressed roots was inhibited and in the same tissues increased cytokinin content was reported [56]. However, the authors surmised that the cytokinin increase was not involved in the growth inhibition, since cytokinins would also inhibit LRI while this did not occur in Cu-stressed roots [75,76]. Vitti *et al.* [60] reported Cd-induced upregulation of a cytokinin turnover gene, *CKX5* (*CYTOKININ OXIDASE* *5*) in *A. thaliana* roots, which resulted in an enlarged root meristem [78]. In other species like green algae, cytokinins were found to restore the Cd-induced inhibition on the photosynthetic capacity [79]. In wheat and soybean a reduction of cytokinin content was reported during Cd stress (Table 1) [80].

## 4. Gibberellins

Gibberellic acids (GAs) were originally identified as a fungal (*Gibberella fujikuroi*) toxin causing unusual shoot elongation of rice plants [81]. They promote germination, elongation growth, flowering and fruit development. GAs are known to move relatively freely from shoots to roots through the phloem [82]. GAs are synthesized in the meristem and accumulate in endodermis cells in the EZ, but no specific transporters are so far reported. Recently, the *Arabidopsis* abscisic acid (ABA) transporter AIT3 (ABA-IMPORTING TRANSPORTER 3) was reported to import GA_3_ into yeast cells, but this has not been confirmed *in planta.* However, it is unlikely that AIT3 is responsible for the GA accumulation in the endodermis since it is mainly expressed in the vascular bundle and developing seeds [82,83].

DELLA proteins modulate the expression of GA-responsive genes by interacting with TFs such as PIFs, ALC and SCL3 (PHYTOCHROME INTERACTING FACTORS, ALCATRAZ and SCARECROW-LIKE 3). In *Arabidopsis* there are five DELLA proteins: GAI, RGA, RGL1, RGL2 and RGL3 [72]. When GID1 (GA INSENSITIVE DWARF1) a, b or c receptors are bound by GA [84,85], they form a complex with DELLA proteins that is subsequently recognized by the SCF^SLY1^ (SLEEPY 1) complex and targeted for degradation by the proteasome [86,87].

GAs enhance root elongation, their primary action site during root growth is the EZ, in which they accumulate (Figure 3E) [82,88,89]. GAs are involved in the regulation of meristem size in *Arabidopsis* by interacting with the cytokinin-auxin antagonism (Figure 3F). When meristem size is set after embryonic development, a decrease in GA biosynthesis results in the expression of the cytokinin-response factor *ARR1*, mediated by DELLA protein RGA, thereby inducing cell differentiation [90]. GA acts as a positive regulator of root growth and meristem size by promoting cell division in the MZ (downstream of auxin) through the degradation of DELLAs [88,89,90]. Additionally, auxin also induces the degradation of these DELLA proteins in the MZ and TZ (Figure 3E) [1,91]. DELLA proteins directly control auxin transport by affecting both transcription and protein stability of PIN auxin transporters (Figure 3G) [90]. During gravitropism, GA accumulation at the (new) lower side of the root results in the PIN2 stabilization that is necessary to transport auxin to the EZ in order to inhibit cell elongation. However, this GA accumulation during gravitropism follows an initial PIN3-mediated auxin accumulation, suggesting that GA and auxin act interdependently [82].

GAs play an important role in the protection against Cd stress, by diminishing the Cd-induced changes (Table 1). Cd exposure causes increased expression of *IRT1*, a transporter that is hypothesized to be involved in the Cd uptake, and this upregulation was suppressed by GAs. Cd also induces nitric oxide (NO) accumulation, which was also reduced by GAs [92]. GAs can also increase mitotic activity, carbohydrate metabolism and the contents of protein and RNA, eventually enhancing Cd tolerance [93].

## 5. Abscisic Acid

Abscisic acid (ABA) is a carotenoid-derived molecule. It plays a role in fruit and leave abscission, germination, control of seed dormancy, bud growth, lateral root outgrowth, stomatal aperture and stress response [94,95]. The involvement of ABA in responses to drought stress is especially well characterized. When the roots sense a decrease in soil water, ABA biosynthesis in the root tips is enhanced and ABA is transported to the leaves where it will stimulate its synthesis in the leave vascular tissues. Enhanced ABA concentrations will induce stomatal closure [96]. Primary root elongation is stimulated and lateral elongation is inhibited by ABA during drought stress [96,97,98]. Basal levels of ABA are required for maintaining normal root growth, however at high concentrations it is a growth inhibitor [95,97,99].

ABA is synthesized both in roots and leaves, with the highest content of physiologically active ABA in the columella root cap and QC cells [100]. Long-distance movement from root to shoot occurs through the xylem and in the inverse direction through the phloem. Known ABA carriers in *Arabidopsis* are ABCG40 and ABCG25 (ATP-BINDING CASSETTE G40 and G25), and are located on the plasma membrane. ABCG40 is proposed to be an importer [101] and ABCG25 an exporter [102]. Recent studies identified two possible ABA transporters in the NRT1/PTR family (NITRATE TRANSPORTER1/PEPTIDE TRANSPORT): AIT1 and AIT3 (ABA-IMPORTING TRANSPORTER 1 and 3) [103,104].

ABA is perceived by multiple receptors, localized in different subcellular compartments that mediate different ABA responses. (1) GTG1 and GTG2 (GPCR-TYPE G PROTEIN) are plasma membrane-localized ABA receptors that function in the regulation of seed germination and seedling growth in *Arabidopsis*, and are negatively regulated by GPA1 (G PROTEIN ALPHA SUBUNIT 1) [72,105]; (2) The PYR/PYL/RCARs (PYRABACTIN RESISTANCE/PYRABACTIN RESISTANCE-LIKE/REGULATORY COMPONENT OF ABA RECEPTORS) are nucleocytoplasmic ABA receptors that are involved in the ABA-regulated seed germination, seedling growth, as well as guard cell movement [106,107,108]. The ABA-bound receptor inactivates PP2Cs (TYPE 2C PROTEIN PHOSPHATASES) by docking within the PP2C active site. Inactivation of PP2Cs leads to accumulation of the active, phosphorylated snRK2s (SNF1-RELATED PROTEIN KINASES), which phosphorylate and thereby activate downstream responses [72]; (3) CHLH (CHELATASE H SUBUNIT) is a chloroplast-localized ABA receptor that mediates the ABA-regulated seed germination, seedling growth and guard cell movement [108]. ABA binding stimulates the removal of transcriptional repressors WRKY40, WRKY18 and WRKY60, thereby activating the expression of ABA responsive TF such as ABI4, ABI5 (ABA INSENSITIVE 4, 5), ABF4 (ABA-RESPONSIVE ELEMENT BINDING FACTOR 4) and MYB2 [72,109].

ABA blocks LRI [110] by upregulating the expression of ICK1/KRP1 (INTERACTOR OF CDC2 KINASE 1/KIP RELATED PROTEIN 1) that inhibits cell division by regulating G1-to-S progression (Figure 3H) [110,111,112]. It is unclear whether auxin and ABA act antagonistically during LRI, although downregulation of KRP levels by auxin supports that notion [110]. During LRP formation *NCED* (involved in ABA biosynthesis) is expressed at the base of the primordium [113]. The ABA-mediated decrease of cell proliferation in neighbouring cells could be essential to define the organ boundaries for a future lateral root (Figure 3H). Once the primordium has emerged, the activation of the LR meristem is controlled by ABA. *ABI1*, *ABI2* and *ABI3* play a role in the ABA-induced LR dormancy in unfavourable soil conditions (Figure 3H) [54,95]. Auxin can stimulate the expression of ABA-responsive genes in LRP by inducing TFs like ABI3 [114] and MYB96 [115,116]. Both *ABI3* and *MYB96* are expressed in developing LRP primordia, thus auxin induction of the TFs can contribute to ABA-mediated meristem activation of the primordia [114,115]. ABA regulates root growth through enhancing auxin signalling, by inducing the expression of *ARF2*, *DR5* and *IAA2* and by inhibiting the transcription of auxin-signal repressors *AXR2/IAA7 and AXR3/IAA17* (Figure 3I) [116]*.* In the root tip ABA also promotes QC maintenance and suppresses cell differentiation in the stem cell niche (Figure 3H) [112]. This ABA-regulated stem cell differentiation in the root meristem requires the function of WOX5 (WUSCHEL-RELATED HOMEOBOX 5), a major regulator of stem cell maintenance [112]. ABA can induce auxin-conjugating enzymes, that inactivate biologically active auxins, under the control of MYB96 (Figure 3I) [117]. ABA also takes part in the cytokinin-mediated regulation of polar auxin transport (PAT). Both ABA and cytokinin can activate ABI4-mediated repression of *PIN1* expression (Figure 3J) [91,118]. ABA has a negative impact on cytokinin content in the roots, by downregulating cytokinin biosynthesis genes and upregulating cytokinin oxidases and dehydrogenases [119]. The cytokinin receptors can also negatively regulate ABA (Figure 3K). Overall cytokinin and ABA appear to have antagonistic roles in controlling the drought response [120].

A rapidly elevated ABA level under stress conditions has been suggested to modulate plant growth and adaptive stress responses [121,122]. Only few reports are available on the metabolic mechanisms through which ABA acts in inducing Cd tolerance (Table 1). ABA signalling is considered an important signal transduction factor during Cd-stress, in which it was found to have a protective role [93,123]. Because of an ABA-mediated decrease of transpiration rate, Cd translocation was reduced in *O. sativa* [124,125]*.* Moreover, ABA can also initiate the production of metal detoxification compounds such as phytochelatins [126,127].

## 6. Ethylene

Ethylene is a gaseous plant hormone that has profound effects on many aspects of plant growth and development, and was first identified in 1901 by Dimitri Neljubov as the active component in illuminating gas, causing premature senescence, abscission and ripening in nearby vegetation [128]. It influences plant growth in dark and light. The triple response phenotype has been used to screen for mutants that are defective in ethylene responses. It is observed in ethylene-treated etiolated *Arabidopsis* seedlings and consists of inhibition of hypocotyl and root elongation, radial swelling of hypocotyl and root cells, and exaggerated apical hook [129]. The highest production rate of ethylene is in meristematic, ripening or stressed tissues. Ethylene dissolves about 14 times better in lipid membranes than in the aqueous phase of cells. Ethylene is involved in seed germination, root initiation and elongation, hypocotyl elongation, flower development, sex determination, abscission, fruit ripening and senescence [130,131].

All five ethylene receptors in *Arabidopsis*, ETR1, ETR2 (ETHYLENE RESPONSE 1 and 2), ERS1, ERS2 (ETHYLENE RESPONSE SENSOR 1 and 2) and EIN4 (ETHYLENE INSENSITIVE 4), are ER-membrane-localized negative regulators of the ethylene signalling pathway [72,132]. In the absence of ethylene these receptors interact with CTR1 (CONSTITUTIVE TRIPLE RESPONSE 1), a MAPKK kinase that represses EIN2 [72,130]. Ethylene signalling begins with binding of ethylene to receptor proteins, thereby inactivating them and releasing EIN2 from its inhibition by CTR1 [72,130,132]. EIN2 controls, either directly or indirectly, the activity of EIN3 and EIL (EIN3-LIKE) TFs, whose targets include *ERF1* (*ETHYLENE RESPONSE FACTOR 1*) and *EDF1/2/3/4* (*ETHYLENE RESPONSE DNA BINDING FACTOR 1/2/3/4*), which will induce the expression of ethylene responsive genes, including ethylene biosynthesis genes to produce more ethylene [119,133,134,135].

EIN2 levels are controlled by SCF^ETP1/2^ (EIN2-TARGETING PROTEIN 1/2) complexes and EIN3/EIL1 levels by SCF^EBF1/2^ (EIN3-BINDING F-BOX 1/2) complexes [136,137]. EBF1/2 F-box proteins are regulated by ubiquitin/proteasome pathway dependent on EIN2 [138], and EBF1/2 mRNAs are degraded by EIN5 [135].

Ethylene is a strong inhibitor of root elongation, LR development and gravitropic responses, it increases the frequency of root waving and stimulates root hair formation [25,139,140]. Ethylene controls these processes by regulating auxin transport within the root tip (Figure 3L) [25,91,139,140]. Ethylene stimulates the expression of auxin biosynthesis genes, and of *AUX1* and *PIN2*, resulting in an increased basipetal auxin transport toward the EZ [23,25,139,140]. In the EZ the synthesis of flavonoids (auxin transport inhibitors) is induced in an *ETR1*, *EIN2* dependent manner [141,142], which contributes to the ethylene-induced auxin accumulation in the EZ that is essential for the ethylene-mediated inhibition of cell elongation (Figure 3L) [23,140,143]. Additionally, ethylene reduces cell expansion capacities by increasing the formation of specific cell wall components, possibly also through the induced auxin accumulation in EZ [144].

Cytokinins stabilize ACS5 and ACS9 enzymes that are involved in ethylene biosynthesis [91]. By stimulating ethylene production cytokinin contributes to the ethylene-induced auxin accumulation that represses cell expansion (Figure 3M) [91]. Su and Howell suggested that cytokinin or cytokinin-induced ethylene signalling is transmitted via an *EIN2*-pathway [145].

Most stress conditions result in higher endogenous ethylene levels [56,93,146,147]. Cd-stress resulted in a fast up-regulation of ethylene responsive genes [147,148]. In *A. thaliana* the Cd-induced enhancement of ethylene production depended on *ACS2* and *ACS6* expression [146]. Ethylene can reverse the inhibition of photosynthesis by Zn, by changing the activity of Photosystem II, the efficiency of photosynthetic nitrogen use and by interacting with the antioxidant metabolism [149] (Table 1). Cu also induces ethylene accumulation [150], however ethylene signalling is probably not involved in the Cu-induced inhibition of root elongation, in contrast to the Al-induced inhibition of root elongation [151]. Lequeux *et al.* [56] reported that ethylene content was not increased in *A. thaliana* after nine days of Cu-stress, while Arteca and Arteca [147] did find an increased ethylene production after 24 h. These contradictory findings could suggest that ethylene is not involved in long-term root responses.

## 7. Jasmonic Acid

Jasmonic acid and its derivatives, collectively referred to as jasmonates, form a group of oxylipin signalling molecules that regulate numerous physiological processes, including wound responses, secondary metabolite synthesis, and defence against biotic and abiotic stresses. In dicots, JA is widely believed to be predominantly effective against necrotrophic pathogens and herbivorous insects, whereas salicylic acid (SA) signalling is typically associated with immunity against biotrophs [152,153,154]. Jasmonates are derived from tri-unsaturated fatty acids that are released from plastids, in which the first part of JA synthesis occurs. After several enzymatic reactions the biosynthesis cycle is translocated to peroxisomes until the bioactive (+)-*7-iso-*JA is formed. Additionally, numerous JA metabolites are formed through conjugation. The most active natural form of jasmonates is Ja-Ile (jasmonoyl-l-*iso*-leucine) [153,154].

The JA receptor COI1 (CORONATINE INSENSITIVE 1) is an F-box protein of the SCF^COI1^ complex [155,156,157,158]. When JA is present the SCF ^COI1^ will bind JAZs (JASMONATE ZIM-DOMAIN PROTEINS) and target them for ubiquitination and subsequent degradation. JAZs are negative regulators of JA-responsive genes because of their interaction with TPL (TOPLESS) via NINJA (NOVEL INTERACTOR OF JAZ), to repress transcription factors such as MYC2 [154,159]. There are 12 JAZ proteins in *A. thaliana* [72,153,158]. JAZ proteins interact with each other, co-repressor proteins, and with other TFs in JA signalling and TFs of other hormone signalling pathways [153].

MYC2 is the most prominent TF in JA signalling, because it regulates the expression of *VSP2* (VEGETATIVE STORAGE PROTEIN 2), an important JA-responsive marker gene. MYC3 and MYC4 are homologous proteins of MYC2 that enhance the MYC2-regulatory effect [153]. MYCs can act as either activator or repressor of distinct JA-responsive genes in *Arabidopsis*. MYCs activate JA-induced root growth inhibition, anthocyanin biosynthesis and oxidative stress tolerance, but represses resistance to pathogens and biosynthesis of auxins [160]. Two members of the NAC TF family, ANAC019 and ANAC055, act as positive regulators of JA-induced expression, downstream of COI1 and MYC2 [161].

JA increases auxin biosynthesis by inducing the expression of biosynthesis genes (Figure 3N). Conversely, auxin attenuates JA signalling by upregulating the *JAZ1* gene [153]. The JAZ1 repressor is an early auxin-responsive gene, and its transcriptional activation by auxin is independent of JA signalling [162]. JA-mediated inhibition of primary root growth is due to the arrest of mitosis [153,158]. This effect occurs via a cross-talk with auxin in which COI1, JAZ10 and MYC2 are involved. MYC2 represses *PLT1* and *PLT2* genes, thereby reducing the size of the RAM [155,160,163]. JA-mediated PIN2 accumulation results in a positive effect of JA on LRI and subsequently LR formation [164]. COI1 is required for the JA-induced signal transduction events in pericycle cells to form LRP [153] and COI1 is possibly also required for the emergence of LRs. The formation of LRs was stimulated by JA mainly at stages I–V, indicating that JA exerted its effects mostly on the initiation of LRP [165]. An antagonistic cross-talk between JA and GA forms the basis of plant growth *versus* plant defence investments of the plant (Figure 3O). MYC2 competes with the RGL DELLA protein to bind JAZ proteins [119]. High levels of JA release MYC2 from JAZs, resulting in the transcription of JA-regulated genes involved in defence and genes encoding RGL (thus suppressing the GA response). In contrast, GA accumulation will lead to the degradation of RGL, resulting in the activation of GA-regulated growth response genes via PIF. Degradation of RGL will release JAZs and they will bind an inhibit MYC2 [153].

Ethylene can inhibit root growth through its own ETR-CTR1 associated pathway, but also through a COI1 and light-dependent, but JA-independent pathway. Additionally, both JAs and ethylene can induce *ERF1* expression [153,166] JAZs can repress expression of ethylene-responsive genes by interacting with EIN3/EIL1 TFs, and high JA concentrations releases JAZ inhibition of EIN3/EIL1 thereby permitting ethylene responses (Figure 3P) [91,130,136].

JAs can neutralize toxic effects of low concentrations of Cu and Cd by inducing the accumulation of phytochelatins, glutathione and carotenoids, which results in an enhanced plant tolerance. Treating *A. thaliana* with Cd and Cu induced jasmonate biosynthetic genes and JA levels were elevated [93,123,150]. In pea plants, increased JAs and ethylene, together with ROS, regulates the induction of pathogenesis-related proteins that protect the plant from Cd-related damages [167]. JA might also interact with ROS signalling as it mediates ROS generation in Cd exposed plants. At high Cd exposure concentrations JAs may be involved in the induction of growth reduction, chlorophyll degradation and inhibition of photosynthesis [123] (Table 1).

## 8. Strigolactones

Strigolactones (SLs) are carotenoid-derived terpenoid lactones, and were first identified as germination stimulants for the parasitic plants *Striga* and *Orobanche* [168,169,170,171]. They stimulate hyphal branching of the symbiotic arbuscular mycorrhizal fungi (AMF) and act as long distance branching factors that suppress growth of preformed axillary buds [172,173].

How SLs are transported in plants is still largely unknown. Experiments in pea, petunia and *Arabidopsis* showed that SLs can move from root to shoot, however not in inverse direction. Possibly linked with the absence of mycorrhization, there is no SL-transporter in *A. thaliana* that is orthologous to the petunia PDR1 (PLEIOTROPIC DRUG RESISTANCE 1) SL transporter and the closest homologue is the ABA transporter ABCG40 [101,168,169,172].

In *Arabidopsis*, the F-box protein MAX2 and the α/β hydrolase protein D14 are SL receptors. The binding of SL to D14 changes its structure, after which MAX2 is triggered to bind and subsequently degrade currently unknown repressors of the SL-signal. A similar mechanism involving MAX2 is described for plant growth promoting effects of karrikins, smoke-derived compounds structurally related to SL and involved in seed germination [172].

SLs are involved in root growth, lateral root formation, root hair elongation, adventitious rooting, stem elongation, secondary growth, leaf expansion, and most prominently, shoot branching [168,169,172,173]. SLs positively regulate PR elongation via MAX2-signaling [169]. SLs increase the cell number and size of the RAM, however, the size of the meristematic cells themselves is reduced significantly. The effects on the RAM are the result of a decrease of the expression of the PIN1, 3 and 7 auxin carriers (Figure 3Q) [168]. SLs are mediators of LR formation with a negative effect under high phosphate and a positive effect under low phosphate conditions, in both cases via MAX2 signalling [174]. Under low phosphate conditions the changes in LR formation in *Arabidopsis* were suggested to be a result of increased auxin sensitivity, mediated via TIR1. Furthermore, *PIN1* expression is decreased by SL, which could also be part of the negative effect on LR formation (Figure 3Q) [168,175]. SLs induce ethylene biosynthesis by stimulating the expression of *ACS2* and both hormones are involved in the regulation of root hair elongation (Figure 3R) [91,174]. Strigolactone and ABA are both derived from carotenoids and their biosynthesis genes are closely related. Also the ABA transporter is the closest SL transporter homologue in *Arabidopsis* [101]. Thus the biosynthesis and transport of these hormones are closely interdependent (Figure 3S). Because of the close relation with ABA, SLs may also be affected when ABA biosynthesis or transport is affected by metal stress. The involvement of SLs in root hair elongation could also be important during drought responses [101,119,171].

## 9. Brassinosteroids

Brassinolide was the first brassinosteroid isolated from *Brassica napus* and since then more than 50 natural analogs, collectively called brassinosteroids (BRs), have been characterized in various plant species [176]. Brassinosteroids are polyhydroxylated triterpenoids essential for plant growth, they participate in seed germination, male fertility, vascular development, fruit ripening, flowering time, senescence and plant response to light, temperature, salt and pathogens among others [91,176,177,178].

Highest BR concentrations are found in young tissues, reproductive organs, seeds, and fruits. BRs do not undergo long-distance transport and thus function as a local signal [179,180]. BRs are perceived by a PM-localized protein, BRI1 (BRASSINOSTEROID INSENSITIVE 1), BRL1 (BRI1-LIKE1) and BRL3. BRL1 and BRL3 are expressed in vascular cells, whereas BRI1 can be found in all types of dividing cells [181]. The binding of BR to BRI1 stimulates the dissociation of the negative regulator BKI1 (BRI1 KINASE INHIBITOR 1) [182]. Subsequently co-receptor BAK1 (BRI1-ASSOCIATED RECEPTOR KINASE 1) or its homologs BKK1 (BAK1-LIKE1) and SERK1 (SOMATIC EMBRYOGENESIS RECEPTOR KINASE 1) can bind to BRI1 [181,183,184].

Subsequently BRI1 phosphorylates BSKs (BR-SIGNALING KINASES), causing it to bind BSU1 (BRI1 SUPPRESSOR 1). The formed complex inactivates BIN2 (BRASSINOSTEROID INSENSITIVE 2), leading to dephosphorylation and activation of the TFs BZR1 (BRASSINAZOLE RESISTANT 1) and BZR2 by PP2A (phosphatase 2A) [181,185,186,187].

Auxin and BR synergistically promote root growth but BR action has been proposed to be subordinate to that of auxin [91,188,189]. BRs promote LRI by increasing acropetal auxin transport in the root through stimulation of *PIN1* and *PIN2* expression (Figure 3U) [190,191,192]. BRI1 signalling is required for normal cell-cycle progression of meristematic cells and maintenance of QC cells, which is central for meristem maintenance in the primary root (Figure 3T) [188,193]. BR-mediated promotion of cell elongation involves activation of cell wall loosening enzymes, such as XETs (XYLOGLUCAN ENDOTRANSFLYCOSYLASES), and the binding of BZR2 to the promoters of a group of *AtCESA* genes (*CELLULOSE SYNTHASE*) that control primary cell wall extension (Figure 3T) [188,194].

In *A. thaliana*, *BRX* (*BREVIS RADIX*) expression is induced by auxin and mildly repressed by BRs [195]. *BRX* positively regulates BR biosynthesis genes [196]. BRX partakes in the cytokinin-auxin regulation of meristem size as a direct target for ARF5/MP and by enhancing *PIN3* expression. At the TZ, cytokinin activates IAA3/SHY2 thereby attenuating the ARF-mediated auxin signalling. As a result the *BRX* expression is restricted and *PIN3* expression decreases, resulting in a reduced auxin flux and thus limited controlled RAM growth (Figure 3V) [197]. BR signalling also interferes with other hormones. ABA is an antagonist of BR signalling (Figure 3W). It represses the expression of BEE (BR-ENHANCED EXPRESSION) TFs that are involved in the BR response in *A. thaliana*. BR signalling is antagonized by SL signalling through the MAX2/D14 dependent degradation of the active TF BZR2 and related proteins (Figure 3W) [172,198,199]. BR is involved in ethylene-induced hyponasty through ROT3. ROT3 is an enzyme that catalyses the conversion of typhasterol to castasterone, a direct precursor of brassinolide that serves as a biologically active form of BR. ROT3 mediates cell expansion via BR, and thus act as a positive regulator of ethylene-induced hyponastic growth [177]. BR stimulates ethylene production by stabilizing ethylene biosynthesis enzymes ACS5 and ACS9. Note that cytokinin had the same effect on ACS5, ACS9 and thus ethylene production [91]. Thus, both BRs and cytokinins contribute to the ethylene-auxin cross talk that controls cell elongation, by stimulating ethylene production (Figure 3M).

In *Brassica napus* and *Lycopersicon esculentum,* BRs reduce the toxic effects of Cd on phytochemical processes by diminishing the damage on photochemical reaction centres and the activity of oxygen evolving centre as well as by maintaining efficient photosynthetic electron transport [200,201,202]. However, in *A. thaliana* BRs do not seem to have the protective effect to Cd stress. Cd exposure triggers the activation of the BR signalling pathway and high BR contents lead to hypersensitivity to Cd (Table 1) [199]. The mechanisms of this sensitivity need further investigation.

## 10. Salicylic Acid

Salicylic acid (SA) is a phenolic compound named after the willow tree (Salix) because the leaves or bark are rich in SA. Historically, chewing leaves or bark of Salix was used against fever, pain and inflammation [203,204]. In 1899, Felix Hoffman, working for the Bayer Company in Germany, made acetylsalicylic acid (aspirin), a synthetic derivate of SA. SA is synthezed from chorismate in chloroplast or from phenylalanine in the cytoplasm via different pathways [203,204,205,206]. SA regulates cell growth, respiration, stomatal aperture, senescence, fruit yield, seed germination, seedling development and thermo-tolerance. It has a crucial role in plant pathogen response, and is involved in responses to abiotic stresses such as chilling, heat, heavy metal toxicity, drought, osmotic stress and salinity [203,204,206].

NRP1 protein (NONEXPRESSOR OF PATHOGENESIS RELATED 1) is as a key component of SA signalling). NPR1, as well as its paralogs NPR3 and NPR4, serve as SA receptor proteins [152]. SA has a lower binding affinity to NPR3 compared to NPR4. SA signalling is connected with NPR1 turnover: NPR3 and NPR4 serve as CUL3 (Cullin 3) E3 ubiquitin ligase adaptors to promote degradation of NPR1 [203]. Elevated SA levels lead to NPR1 monomerization and the translocation of NPR1 monomers to the nucleus, where they induce defence gene expression. Thus NPR1 is not only a SA receptor, but also an effector [203]. NRP1 is also involved in the downregulation of the *ICS1* gene, which is responsible for the conversion of chorismate to ultimately SA, thus forming a negative feedback loop [204]. Roots of SA-treated plants were relatively short, with the root hairs localized close to the root tip, and had accelerated LR formation [207].

The TF MYB96 regulates synergism between SA and ABA [208]. Both Ethylene and SA activate pathogen response gene expression through multiple points of convergence as AP2/ERF (APETALA 2), ERF1, ORA59 (OCTADECANOID RESPONSIVE ARABIDOPSIS 59), and CEV1 (CONSTITUTIVE EXPRESSOR OF VSP1) [209]. In plant immunity, ET-JA is generally thought to induce necrotroph resistance while ET antagonizes SA-mediated biotroph resistance. However, depending on the infection strategy of the pathogen, ethylene can interact both positively and negatively with SA, depending on the infection strategy of the invading pathogen (Figure 3X) [210,211]. In plant defence against pathogen attacks, the crosstalk between JA and SA is very important (Figure 3Y). Low levels of SA and JA act synergistically. The SA receptor and effector NPR1 is involved in suppressing JA-inducible genes, and the JA-signalling proteins MPK4 (MITOGEN-ACTIVATED PROTEIN KINASE 4), together with SSI2 (SUPPRESSOR OF SA INSENSITIVITY 2) and COI1, are negative regulators of SA-mediated defence [204]. A common element in SA and JA-mediated signalling pathways is the transcription factor WRKY70, which activates SA-induced genes downstream of NPR1 and inhibits JA-responsive genes [212].

SA can act as a direct scavenger of hydroxyl radicals [149] that can be formed during metal stress. SA application significantly improved tolerance against Cd- stress in *Phaseolus aureus* and *Vicia sativa* by increasing antioxidative enzymes and decreasing H_2_O_2_ accumulation [213]. SA reduced Cd uptake, improved photosynthetic capacity, and enhanced antioxidative activities in *Cannabis sativa* [214]. Cd stress induced endogenous SA accumulation in pea, maize and *Arabidopsis* [215,216,217]. In *Arabidopsis* the high SA levels increased Cd-induced plant growth retardation (Table 1) [217].

## 11. Conclusions

The underlying mechanisms of root developmental reactions to metal stress remain largely to be discovered. It is clear that the complexity of hormone synthesis, signal transduction, perception and cross-talk creates networks (Figure 3) that allow for multiple possible interaction points by which metal stress can interfere with normal root developmental programs (Table 1). *Arabidopsis thaliana* is still the preferred organism for discovering molecular mechanisms, given the large collection of mutants and transformants, and the ease of *in vitro* growth, combined with macroscopic and microscopic observation and quantification of root growth. This review provides a reference to current knowledge on gene networks, which can be used for studying the action of plant hormones or disturbance of normal processes resulting in root growth alterations under abiotic stress, and metal stress in particular.

**Table 1:** Effects of metal stress on phytohormone pathways.

Hormone	Effect	Plant
Auxin	Cu inhibits meristematic cell division by decreasing *PIN1* expression [56,57]	*A. thaliana*
	Al inhibits root elongation via *PIN2* and *AUX1* [58]	
	Cd altered *AUX1* and *PIN* expression; *pin2-1* mutant fails to show Cd-induced increase in LR density [59]	
Cytokinins	Increased cytokinin content in inhibited primary root during Cu stress [56]	*A. thaliana*
	Cd induced increased cytokinin content via CKX5 [60,78]	*A. thaliana*
	Cd decreased cytokinin content [80]	*w*heat, soybean
	Restore Cd-induced inhibition on photosynthesis [79]	*Chlorella vulgaris*
Gibberellins	Protect by diminishing Cd induced changes [92,93]	*A. thaliana*
Abscisic acid	Reduces Cd translocation by decreasing transpiration [124,125]	*O. sativa*
	Initiate phytochelatin production [126,127]	Potato
Ethylene	Cd upregulates ethylene responsive genes, via ACS2 and ACS6 [146]	*A. thaliana*
	Reverse Zn-mediated Inhibition of photosynthesis [149]	
Jasmonic acids	Elevated JA levels induce accumulation of phytochelatins, glutathione, carotenoids to enhance tolerance to low Cu and Cd [93,123,150]	*A. thaliana*
Brassinosteroids	Reduce toxic effects of Cd on phytochemical processes [200,201,202]	*Brassica napus*, *Lycopersicon esculentum*
	Hypersensitivity to Cd [199]	*A. thaliana*
Salicylic acid	Improves Cd tolerance by increasing antioxidative enzymes and decreasing H_2_O_2_ accumulation [213]	*Phaseolus aureus*, *Vicia sativa*
	Reduces Cd uptake and enhance antioxidative activities [214]	*Cannabis sativa*
	Cd stress induced SA accumulation [215,216,217]	Pea, maize, *A. thaliana*
